# Supplementary material for: Thymocyte Development of Humanized Mice Is Promoted by Interactions with Human-Derived Antigen Presenting Cells upon Immunization
Source: Int J Mol Sci. 2023 Jul 20;24(14):11705. doi: 10.3390/ijms241411705 (PMC10380196; doi:10.3390/ijms241411705)
Supplement: Supplementary file 1 [file ijms-24-11705-s001.zip › Supplemental Video legend.pdf]

Supplemental Video legend

Supplemental Video S1

Time-lapse imaging of human thymocytes expressing Venus in the thymus of IBMI-huNSG mice with a time interval of 15 sec; scale bar, 40 µm. Green and blue represent thymocytes and collagen fiber visualized by second harmonic generation (SHG), respectively.

Supplemental Video S2

Time-lapse imaging of human thymocytes expressing Venus in the cortex of IBMI-huNSG mice with a time interval of 15 sec; one scale, 7.5 µm. Green and blue represent thymocytes and collagen fibers visualized by SHG, respectively.

Supplemental Video S3

Time-lapse imaging of human thymocytes expressing Venus in the medulla of IBMI-huNSG mice with a time interval of 15 sec; one scale, 7.5 µm. Green and blue represent thymocytes and collagen fibers visualized by SHG, respectively.

Supplemental Video S4

Time-lapse imaging of human thymocytes expressing Venus in the cortico-medullary junction of IBMI-huNSG mice with a time interval of 15 sec. one scale, 7.5 µm. Green and blue represent thymocytes and collagen fibers visualized by SHG, respectively.

Supplemental Video S5

Time-lapse imaging of human thymocytes expressing the Rap affinity probe within the thymus of IBMI-huNSG mice with a time interval of 20 sec. scale bar, 30 µm.
